# The fortune cookie flap for aesthetic reconstruction after chest keloid resection: a small case series

**DOI:** 10.1186/s13019-018-0713-x

**Published:** 2018-04-19

**Authors:** Tae Hwan Park, Jang Won Lee, Chan Woo Kim

**Affiliations:** Department of Plastic and Reconstructive Surgery, CHA Bundang Medical Center, CHA University, 59 Yatap-ro, Bundang-gu, Seongnam, Gyeonggi 13496 Republic of Korea

**Keywords:** Keloid, Fortune cookie, Keystone, Perforator, Flap

## Abstract

**Background:**

Generally, the recurrence rate of keloids is unacceptably high after surgical excision alone. Nevertheless, surgical reduction of keloids is inevitable in many cases. The reconstruction of extensive soft tissue defects following complete keloid resection is challenging to surgeons. In this study, we present our clinical experience using a novel fortune cookie flap for treating chest keloids. This flap provides an excellent surgical option that maintains natural appearance with minimal donor-site morbidity.

**Methods:**

We retrospectively reviewed the data from 3 consecutive cases of reconstruction using the fortune cookie flap following resection of chest keloids between March and December, 2017.

**Results:**

Successful reconstructions were performed without any major complications. The mean dimensions of the reconstructed defect were 5.0 × 4.2 cm, while the mean dimensions of the flap were 7.7 × 5.7 cm.

**Conclusions:**

Owing to its simplicity, reliability, versatility, minimal morbidity and excellent aesthetics, the fortune cookie flap is as an excellent option for reconstruction following complete keloid resection on the chest.

**Electronic supplementary material:**

The online version of this article (10.1186/s13019-018-0713-x) contains supplementary material, which is available to authorized users.

## Background

Generally, the recurrence rate of keloids is unacceptably high after surgical excision alone. Nevertheless, surgical reduction of keloids is inevitable in many cases. The reconstruction of extensive soft tissue defects following complete keloid resection is challenging to surgeons. Specifically in a chest keloid, the tissue mobility is relatively limited; therefore, it is generally accepted that reconstruction after resection of chest keloids is a great burden to surgeons [1]. For this reason, some physicians prefer intralesional excision to preclude any surgical defect that cannot be closed primarily and therefore requires a second surgical procedure, such as flap surgery or skin grafting [2]. On the other hand, skin grafting in patients with keloid is a great concern to both the patient and physician because there is a significant risk of the original keloid recurring and of new keloid formation at the distant donor site.

The keystone flap is a gaining popularity because of its versatility in covering various defects, while offering stable vascularity of the perforator flaps and a relatively easy harvest. Very recently, the authors have presented a series of aesthetic reconstruction after resection of retroauricular keloids with the traditional keystone flap [3]. However, the traditional keystone flap cannot be successfully used in every anatomical region, even in sizeable defects following complete keloid excision. In this study, we present our clinical experiences using a novel fortune cookie flap for patients with resected chest keloid.

## Methods

### Data collection

We reviewed all patients who underwent the fortune cookie flap for surgical defect following complete keloid resection in 2017. This study was approved by the Institutional Review Board of the CHA University and conducted in accordance with the Declaration of Helsinki. A comprehensive review of the basic demographic data, medical histories, wound sites and sizes, wound culture results and complications was performed. Appropriate statistical tests were used for continuous univariate analysis and are given as means ± standard deviation [SD].

### Operative technique

**Fig. 1:**
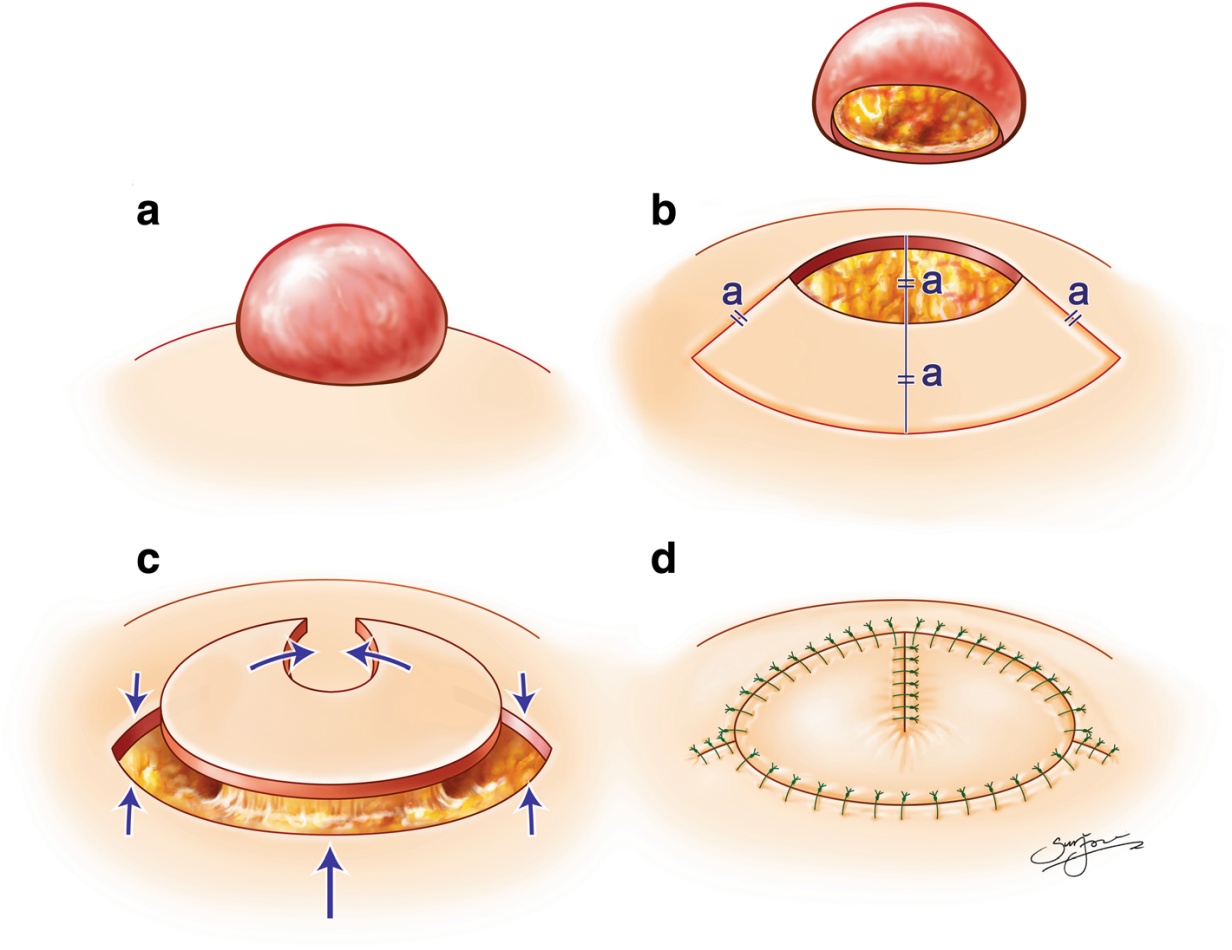
Schematic illustration of a fortune cookie flap, modified from the keystone flap, to cover the surgical defect following complete keloid resection or debridement of infected wounds

In this study, we applied the new fortune cookie flap to cover the surgical defect following complete keloid resection or debridement of infected wounds. The defect was measured intraoperatively in two dimensions, with the longest dimension being the length and its perpendicular axis measurement, the width. We then proceeded with the flap design. As shown in Fig. 1b, the width of the wound “a” is equal to the width of the keystone arc. To effectively cover the defects, we began with complete excision including removal of proliferating core collagen (Fig. 1a, b). After complete resection, each patient underwent immediate reconstruction. The flap was incised along the entire design. The flap was fully elevated while preserving the central “hot spot” including the perforators. Then, the 2 limbs and margins adjacent to the wound were conjoined to cover the defect (Fig. 1c). As illustrated in Fig. 1d, the conjoined flap is a sector shape with a suture line from the centre, giving it the appearance of a fortune cookie.

## Results

We identified and reviewed 3 patients (2 men and 1 woman; mean age, 27 years who underwent the fortune cookie flap procedure for chest keloid resection and reconstruction. The average wound dimensions were 5.0 cm × 4.2 cm, and the average flap size was 7.7 cm × 5.7 cm. Among them, 2 cases were referred from another hospital due to wound disruption with methicillin resistant *Staphylococcus aureus* (MRSA) infection after surgical resection followed by purse-string suture or primary closure (Additional file 1).

**Fig. 2:**
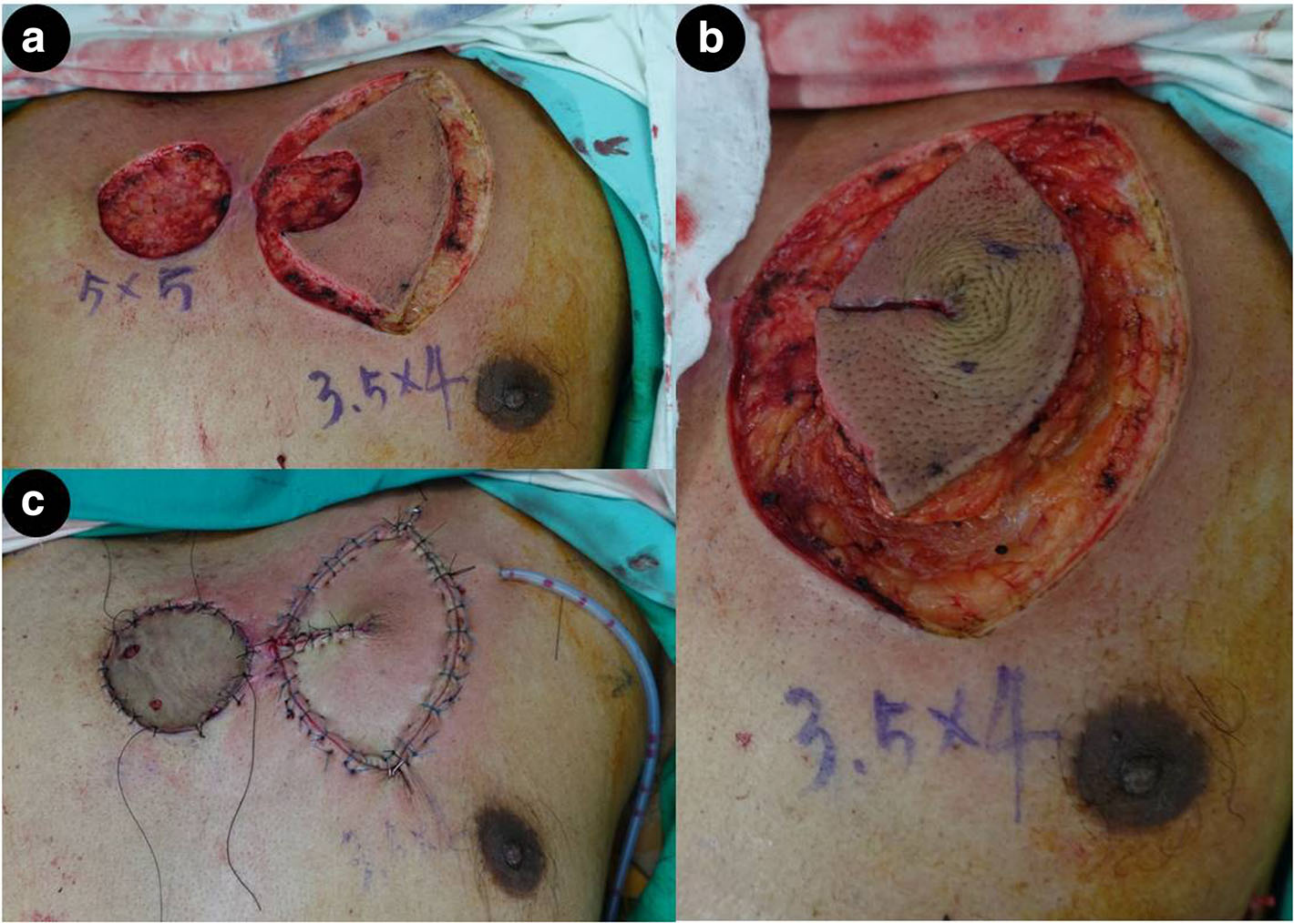
**a** Intraoperative view of 2 defects, measuring 5.0 × 5.0 cm and 3.5 × 4.0 cm, after resection of an infected wound. **b** A 3.5 × 4.0 cm defect on the left side of the anterior chest wall was reconstructed using the fortune cookie design perforator island flap. **c** Final closure following inset of the perforator flap and split thickness skin graft

**Fig. 3:**
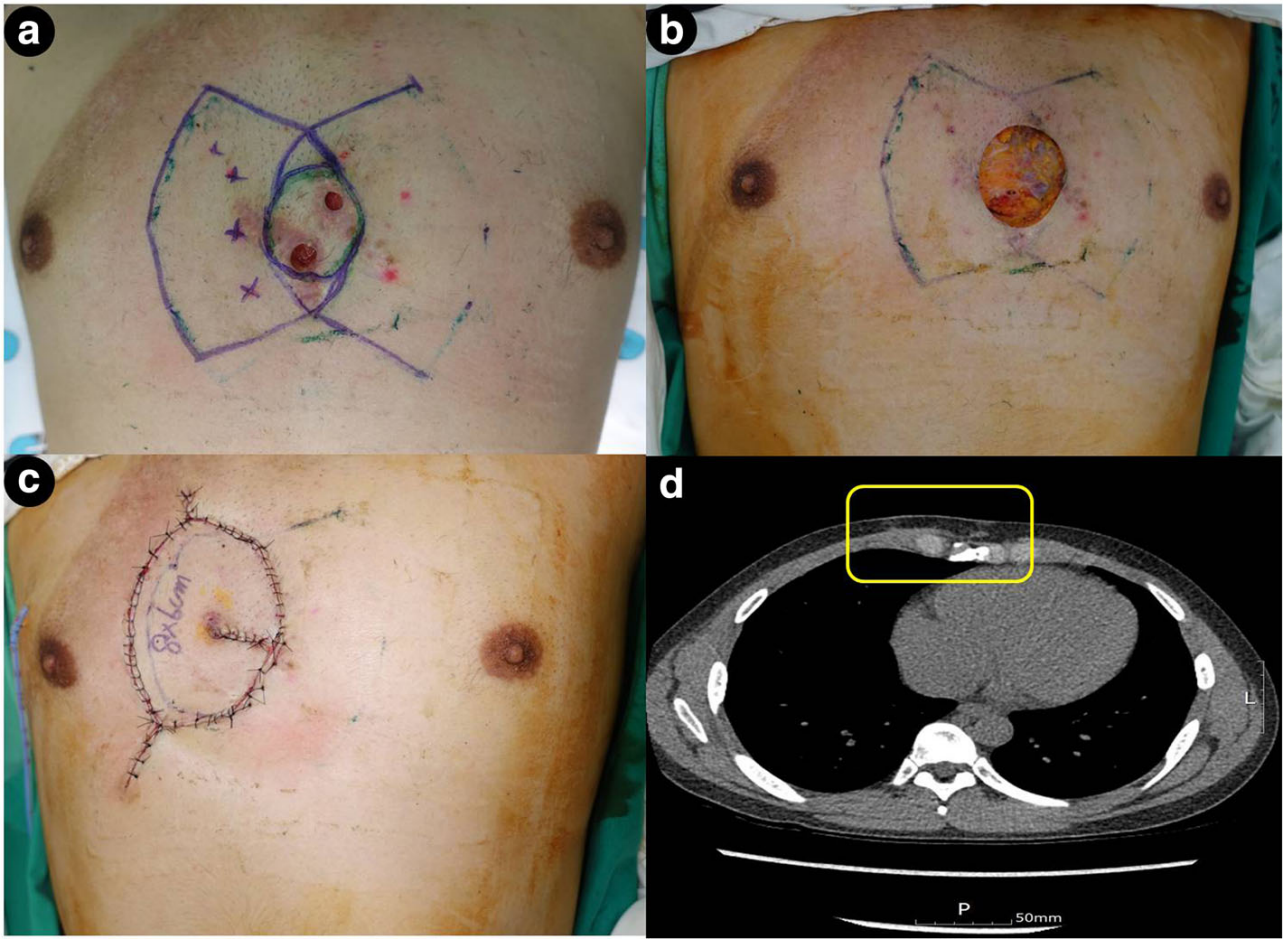
**a** A 29-year-old man with wound dehiscence following a keloid excision with direct primary closure on the anterior chest wall, performed at another clinic. Marking of cutaneous perforators, identified using a handheld Doppler preoperatively, and design of the keystone flap. The wound was infected with methicillin resistant *Staphylococcus aureus* (MRSA). **b** Intraoperative view of a defect, measuring 5.5 × 5.0 cm, after resection of an infected wound. **c** Immediate postoperative result of the fortune cookie design perforator island flap and **d** Computed tomography scan in axial plane at 2 months’ follow-up

The mean operation time from surgical incision to total flap closure was 30 min. The postoperative courses were uneventful. No patient experienced partial or total flap loss or underwent any additional surgery. The representative cases are shown in Figs. 2 and 3.

## Discussion

Once the surgical resection of keloids is indicated, the selected reconstruction method is dependent on the surgeon’s preference and postoperative adjuvant therapy. If radiation therapy is scheduled postoperatively, flap surgery is better than skin grafting. This is because physicians can easily determine when to initiate radiation therapy when performing flap surgery. On the other hand, it is difficult to determine when to initiate radiation in the case of a skin graft because grafted, thin skin has a relatively weak vascularity during early wound healing and early radiation therapy can cause secondary wound problems [4]. Therefore, flap surgery is considered better than skin grafting in many cases of keloid resection. Nevertheless, traditional perforator flap surgery requires tedious perforator dissection and inadvertent injury to perforators leading to flap congestion or necrosis can be encountered [5]. Minimising perforator dissection to prevent perforator injury causes venous congestion and uneven tension distribution, with especially high tension furthest away from the perforator.

Despite growing interest in the traditional keystone flap, the use of this flap for reconstruction after keloid resection is extremely rare. Recently, there have been geometric controversies over the tension distribution with skin paddle expansion of the traditional keystone flap. In using this flap, the possible expansion and advancement has limits and double, opposing keystone flaps are required to repair some large defects [6, 7].

**Fig. 4:**
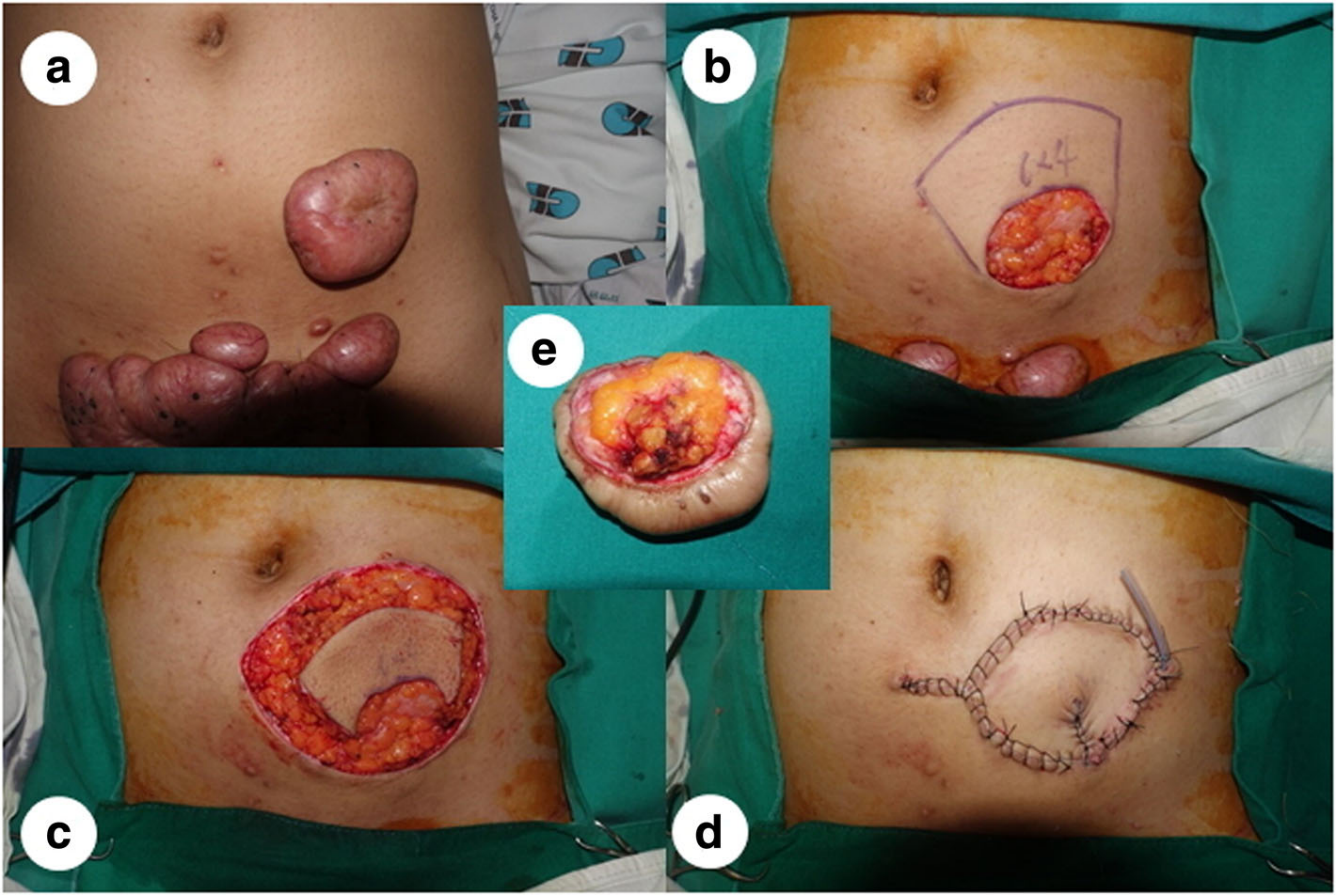
**a** A 21-year-old woman with a large keloid on the lower abdomen, experiencing significant discomfort and severe pain caused by a belt. **b** A 6.0 × 4.0 cm defect following resection of the keloid. **c** A keystone flap designed for reconstruction on the upper side of the defect because of a lack of surrounding tissue laxity and quality, with other keloids on the lower side. **d** Immediate postoperative result of the fortune cookie design perforator island flap with V-Y closure of the donor site with a drain in situ. **e** No remnant proliferating collagen core on the surgical resection margin is shown, which is related to local recurrence

Even though further follow-up is needed, our fortune cookie flap is also effective for MRSA infected open wounds following chest keloid resection. Moreover, the fortune cookie flap is more time-efficient than any other local or distant flaps and can be successfully used in other anatomical locations (Fig. 4). Our average operation time was 30 min, and the total time did not vary much depending on the location. Especially in a perforator-rich area such as the abdomen, re-elevation of a previously-harvested fortune cookie flap, even after prior flap failure, or elevation of new fortune cookie flap using adjacent tissue is always a feasible and reliable option. Based on our experience of this flap in cases other than keloids, the failure rate is extremely low.

Our study has several limitations. This is a small retrospective case series having inherent limitations. Our sample size of 3 is relatively small, and further study of a greater number of cases will aid revealing other strengths or downsides of this technique. In addition, as we did not provide long-term follow up of these cases; further follow-up with the addition of more patients is required.

## Conclusions

Our novel fortune cookie flap provides an excellent surgical option for reconstruction after chest keloid resection that maintains a natural look with minimal donor-site morbidities.

## Additional file

Additional file 1: Previous treatment history at other hospital. (**A**) A 31-year-old man with a keloid on the anterior chest wall. (**B**) The patient had a 7.0 × 3.0 cm defect after resection of a keloid. (**C**) He underwent wound closure using a traditional subcuticular purse-string suture at the other clinic. (**D**) 5 weeks after surgery, the wound was dehisced and methicillin resistant *Staphylococcus aureus* (MRSA) was identified in the wound. (JPEG 84 kb)

## Abbreviations

MRSA: methicillin resistant *Staphylococcus aureus*
SD: standard deviation
