# Supplementary figures and images for: The fortune cookie flap for aesthetic reconstruction after chest keloid resection: a small case series
Source: J Cardiothorac Surg. 2018 Apr 19;13:31. doi: 10.1186/s13019-018-0713-x (PMC5907744; doi:10.1186/s13019-018-0713-x)

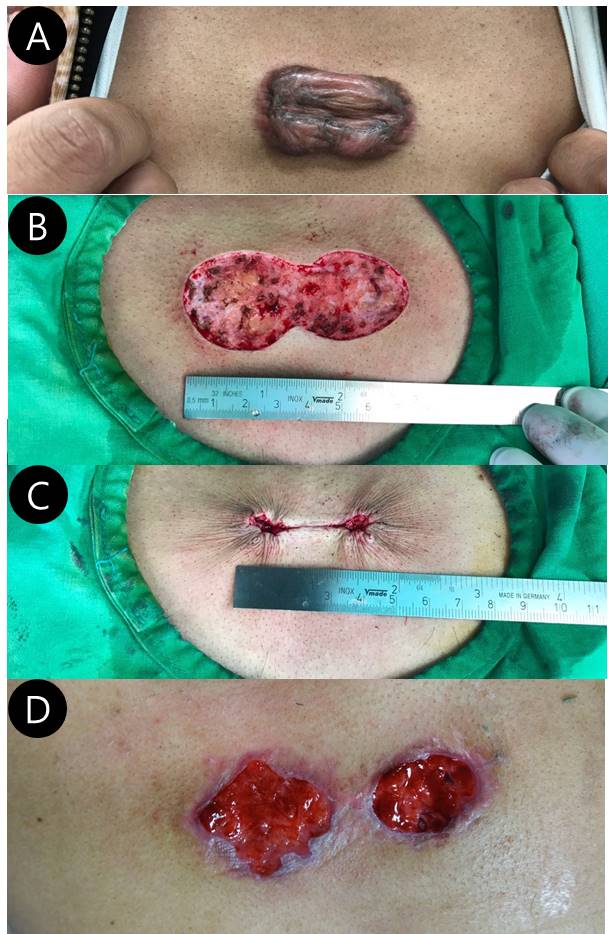

Supplement: Supplementary file 1 — Previous treatment history at other hospital. (A) A 31-year-old man with a keloid on the anterior chest wall. (B) The patient had a 7.0 × 3.0 cm defect after resection of a keloid. (C) He underwent wound closure using a traditional subcuticular purse-string suture at the other clinic. (D) 5 weeks after surgery, the wound was dehisced and methicillin resistant Staphylococcus aureus (MRSA) was identified in the wound. (JPEG 84 kb) [file 13019_2018_713_MOESM1_ESM.jpg]
